# FD-DEIM: efficient and robust detection of small, irregular foliar lesions in the field

**DOI:** 10.3389/fpls.2026.1743266

**Published:** 2026-03-27

**Authors:** HuanYu Zeng, Jiang Tao

**Affiliations:** 1 College of Engineering Science and Technology, Shanghai Ocean University, Shanghai, China; 2 College of Information Technology, Shanghai Ocean University, Shanghai, China

**Keywords:** in-field foliar disease detection, irregular lesion localization, lightweight foliar disease detection, multi-scale feature fusion, small-lesion detection

## Abstract

The automated detection of early-stage foliar diseases in open fields is severely constrained by two theoretical bottlenecks: the physical erasure of high-frequency spatial details during downsampling and the topological mismatch between rigid bounding boxes and irregular lesion morphologies. To systematically overcome these structural limitations, we propose FD-DEIM, a lightweight detection framework optimized for fine-grained feature preservation and dynamic boundary adaptation. The architecture introduces four functionally distinct mechanisms: (1) FD-SRFD, an attention-enhanced stem that anchors sub-pixel coordinates via slicing operations in the initial feature extraction stage, preventing the annihilation of microscopic high-frequency details; (2) DRFD, a deep robust downsampler deployed in the network neck, utilizing a parallel multi-path design to protect high-fidelity spatial features from semantic drift during dimension reduction; (3) FD-Block, an ultra-lightweight fusion module that leverages Partial Convolutions to eliminate computational redundancy while efficiently aggregating multi-scale semantics; and (4) D-FINE, a dynamic offset decoder that forces sampling points to actively deform and tightly hug non-convex, organic fungal contours, fundamentally resolving geometric inductive bias. To ensure ecological validity, we also introduce the RTFD dataset, utilizing generative style transfer to simulate extreme meteorological stressors. Extensive evaluations demonstrate that FD-DEIM achieves a superior AP@50 of 0.667. Crucially, by maintaining an unbroken spatial coordinate preservation chain, the model achieves an *AP_S_* of 0.391 for microscopic targets, a 31.6% relative improvement over the state-of-the-art YOLOv13n. Operating at a minimal computational cost of 7.67 GFLOPs with a 307 ms inference latency on an RK3576 edge CPU, FD-DEIM establishes a new Pareto frontier between rigorous edge-computing constraints and high-fidelity microscopic detection, facilitating proactive precision intervention in agriculture.

## 1 Introduction

Agriculture constitutes the essential component of global economic stability and food security. However, agricultural productivity is increasingly compromised by the proliferation of phytopathogens. The incidence of major foliar diseases, such as bacterial blight and late blight, has intensified due to climate change, which alters temperature and humidity profiles favorable for pathogen propagation (Ristaino et al., 2021). In China alone, the forecasted area of crop infestation reached 2.33 billion mu in 2024, threatening over 70% of major production regions (MARA, 2024). Consequently, unmitigated disease outbreaks could result in global yield losses estimated at 20–40% annually, translating to an economic deficit of approximately $220 billion (Dolatabadian et al., 2025; FAO, 2025).

Traditional disease monitoring paradigms rely heavily on manual visual inspection. This approach is intrinsically labor-intensive and prone to inter-observer variability (Shoaib et al., 2025). Furthermore, demographic shifts in developing nations, characterized by an aging rural workforce and urbanization, render manual scouting increasingly unsustainable. These challenges necessitate the deployment of automated, high-precision detection systems capable of operating in unstructured field environments. While Deep Learning (DL) has achieved remarkable success in controlled settings, deploying these models in open-field precision agriculture faces the persistent “Lab-to-Field” gap. Current state-of-the-art lightweight detectors, particularly the YOLO series (Fuentes et al., 2017; Lei et al., 2025), encounter three systemic limitations when applied to phytopathology:

- Feature Erasure in Microscopic Lesions: Early-stage lesions often occupy fewer than 32×32 pixels. Standard Convolutional Neural Networks (CNNs) utilize aggressive downsampling (e.g., stride-2 convolutions) to expand the receptive field. According to the sampling theorem, this operation acts as a low-pass filter, irrevocably discarding high-frequency spatial details essential for identifying tiny bacterial spots (Zhou, 2020).
- Mismatch of Rigid Anchors and Amorphous Boundaries: Unlike rigid objects (e.g., vehicles), fungal lesions exhibit highly irregular and non-convex morphologies. Anchor-based or static-grid detectors approximate these organic shapes with fixed bounding boxes, leading to poor Intersection over Union (IoU) and inaccurate localization of lesion margins.
- Environmental Interference: Field images are plagued by variable illumination (e.g., specular reflections, shadows) and background clutter (e.g., soil, weeds). Generic backbones lack specific attention mechanisms to suppress these non-target textures, resulting in high False Positive rates.

To systematically address these challenges, this study introduces FD-DEIM (Foliar Disease DETR with Improved Matching), a lightweight detector engineered specifically for the robust identification of small, irregular lesions. The architecture departs from the standard YOLO design paradigm by integrating a detail-preserving feature pathway with a dynamic detection head. Specifically, we redesigned the backbone’s initial stem using the FD-SRFD module and introduced a Deep Robust Feature Downsampling (DRFD) mechanism in the neck. These components utilize slicing operations and parallel processing to retain spectral fidelity during resolution reduction. For feature fusion, the proposed FD-Block leverages partial convolutions to aggregate multi-scale contexts efficiently. Critically, we adopt the D-FINE decoder (Peng et al., 2024), which predicts dynamic sampling offsets to align the receptive field with irregular lesion boundaries, thereby overcoming the limitations of fixed grids.

Beyond architectural innovations, data scarcity remain bottlenecks. We constructed the RTFD (Rice and Tomato Foliar Disease) dataset, utilizing SaMam style transfer (Liu et al., 2025) to simulate extreme meteorological conditions (e.g., rain, snow, high-contrast light). This data-centric approach enhances the model’s invariance to environmental perturbations. The primary contributions of this work are summarized as follows:

1. Ecologically Valid Dataset Construction: We introduce the RTFD dataset, augmented via SaMam style transfer to encompass diverse illumination and weather scenarios, establishing a robust benchmark for in-field diagnosis.
2. Detail-Preserving Architecture Design: We propose the FD-DEIM framework, which integrates DRFD, FD-SRFD, and FD-Block. These modules reduce the loss of fine-grained features during downsampling, enabling the precise detection of microscopic lesions.
3. Validation of Dynamic Localization for Non-convex Lesions: By adapting the state-of-the-art D-FINE decoder to the agricultural domain, we demonstrate that its offset-aware sampling mechanism fundamentally resolves the localization inaccuracies of rigid anchors for irregular foliar lesions, leading to a significant improvement in AP@75.
4. Great Performance with Edge Viability: Extensive evaluations on PlantDoc and RTFD demonstrate that FD-DEIM outperforms YOLOv13n by significant margins (e.g., +7.8% AP@50 on RTFD). Furthermore, deployment tests on the Rockchip RK3576 platform confirm its feasibility for real-time edge applications.

## 2 Related work

Plant diseases pose significant risks to global food security, necessitating timely and precise intervention. Traditional reliance on manual visual inspection is constrained by high labor intensity and susceptibility to subjective diagnostic errors (Shoaib et al., 2025). Consequently, Deep Learning (DL) based computer vision has emerged as a critical tool for automated, high-throughput disease diagnosis in precision agriculture.

Existing research has explored various imaging modalities for disease detection. While multi-modal fusion (e.g., spectral and thermal) offers rich pathological data (Khan et al., 2025), substantial hardware costs limit their scalable deployment. Thus, RGB-based lightweight models remain the standard for edge applications (Bhosale et al., 2023). Methodologically, the field has evolved from hand-crafted descriptors to end-to-end learning (Devi and Srinivasan, 2020). However, given the scarcity of annotated agricultural datasets, recent studies emphasize the necessity of transfer learning and domain-specific preprocessing strategies (Manavalan, 2022). For instance, cross-domain architectures like Thoracic-net (Bhosale et al., 2024) demonstrate that effective identification in limited-data regimes requires not merely deeper networks, but advanced feature fusion mechanisms capable of capturing subtle semantic details—a requirement shared by both medical and agricultural image analysis.

For real-time applications, the YOLO series (Fuentes et al., 2017) is widely adopted due to its inference efficiency. Despite optimizations in recent iterations like YOLOv11, architectural constraints remain when applied to phytopathology. Variants such as RDL-YOLO (Zhang et al., 2025) attempted to enhance context via receptive field expansion, yet they retain the fundamental limitations of strided convolutions. Aggressive downsampling inevitably erodes high-frequency spatial details (Lu et al., 2023), causing microscopic, early-stage lesions to be missed. Furthermore, standard anchor-based mechanisms struggle to regress the irregular, non-convex boundaries typical of fungal infections, while lightweight backbones often lack sufficient noise suppression in complex field environments (Li et al., 2025).

To bridge these gaps, we propose FD-DEIM. Unlike RDL-YOLO (Zhang et al., 2025), which primarily focuses on receptive field expansion, our approach explicitly addresses the feature vanishing problem via a detail-preserving stem and decoupled feature blocks. This ensures the retention of microscopic visual cues lost by standard downsampling. Furthermore, in contrast to the rigid anchor-based assignment in standard YOLO baselines (Fuentes et al., 2017), FD-DEIM employs a dynamic, label-aware matching strategy. This allows for adaptive boundary regression of irregular lesions, effectively balancing detection precision for small targets with the computational constraints of edge devices.

## 3 Materials and methods

This section focuses on introducing the datasets used in the study, data augmentation techniques, evaluation metrics, experimental parameters, and the key improvements in FD-DEIM.

### 3.1 The datasets

To rigorously evaluate the proposed FD-DEIM model, a dual-dataset validation strategy was employed. This approach was designed to assess both the model’s robustness against specific, challenging environmental conditions and its generalization capability on a complex, public benchmark. This strategy directly addresses the “Lab-to-Field” gap, a critical barrier to the practical deployment of detection models in real-world agriculture. The datasets include our novel Rice and Tomato Foliar Disease (RTFD) dataset and the publicly available PlantDoc dataset.

#### 3.1.1 The RTFD dataset: a novel in-field foliar disease dataset

The RTFD dataset was constructed to provide a focused, high-quality corpus of in-field imagery for model training and validation. Primary data acquisition was conducted at Donghai Farm, Shanghai, from August 1 to August 30, 2024, under natural field weather conditions (ranging from sunny to overcast) to capture realistic illumination variations prior to any synthetic augmentation, utilizing a Hikvision DS-2CD3746F(D)WDA3 F-IZS camera. The distance between the camera and the leaves is 50cm. This collection features two economically significant local crops—rice and tomato—captured under authentic field conditions. To complement the self-acquired data, we integrated high-quality foliar disease samples from public repositories: specific Late Blight and Healthy instances were selected from the Dataset of Tomato Leaves (Taiwan) (Chuang and Huang, 2020), while Bacterial Leaf Blight, Brown Spot, and Healthy Rice Leaf classes were sourced from the Rice Leaf Bacterial and Fungal Disease Dataset (Bangladesh) (Islam, 2023). In total, the dataset comprises 920 images from Donghai Farm, 204 from Taiwan, and 604 from Bangladesh, effectively spanning diverse Asian climatic zones.

**Figure 1:**
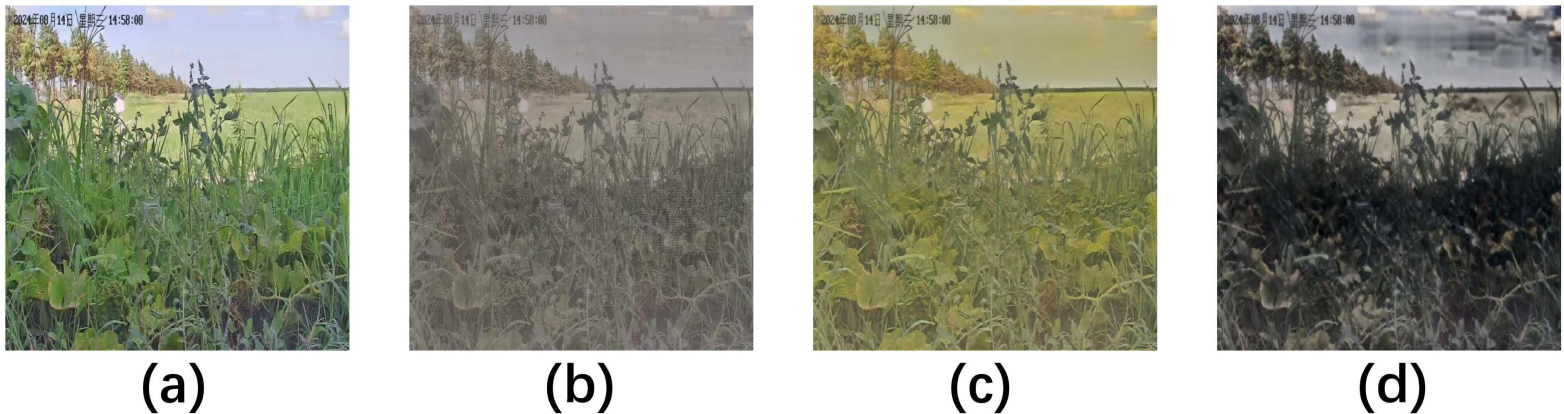
The RTFD dataset is enhanced based on SaMam. **(a)** original; **(b)** rainy and snowy; **(c)** high-light; **(d)** low-light.

To mitigate the challenge of limited data diversity and to specifically evaluate model robustness against standard environmental variables known to degrade performance (Khan et al., 2025), we employed SaMam, a novel style transfer methodology (Liu et al., 2025), for data augmentation. This marks the first application of this technique for simulating agro-environmental conditions. Pre-trained style representations corresponding to rainy/snowy weather, high-intensity light, and low-light conditions were applied to the original images (**Figure 1**). Critically, this augmentation strategy was exclusively applied to the self-acquired dataset; the external public datasets were excluded from this process to prevent the introduction of stylistic bias. This targeted approach facilitated the construction of a dataset capable of simulating key environmental stressors encountered during in-field deployment.

**Figure 2:**
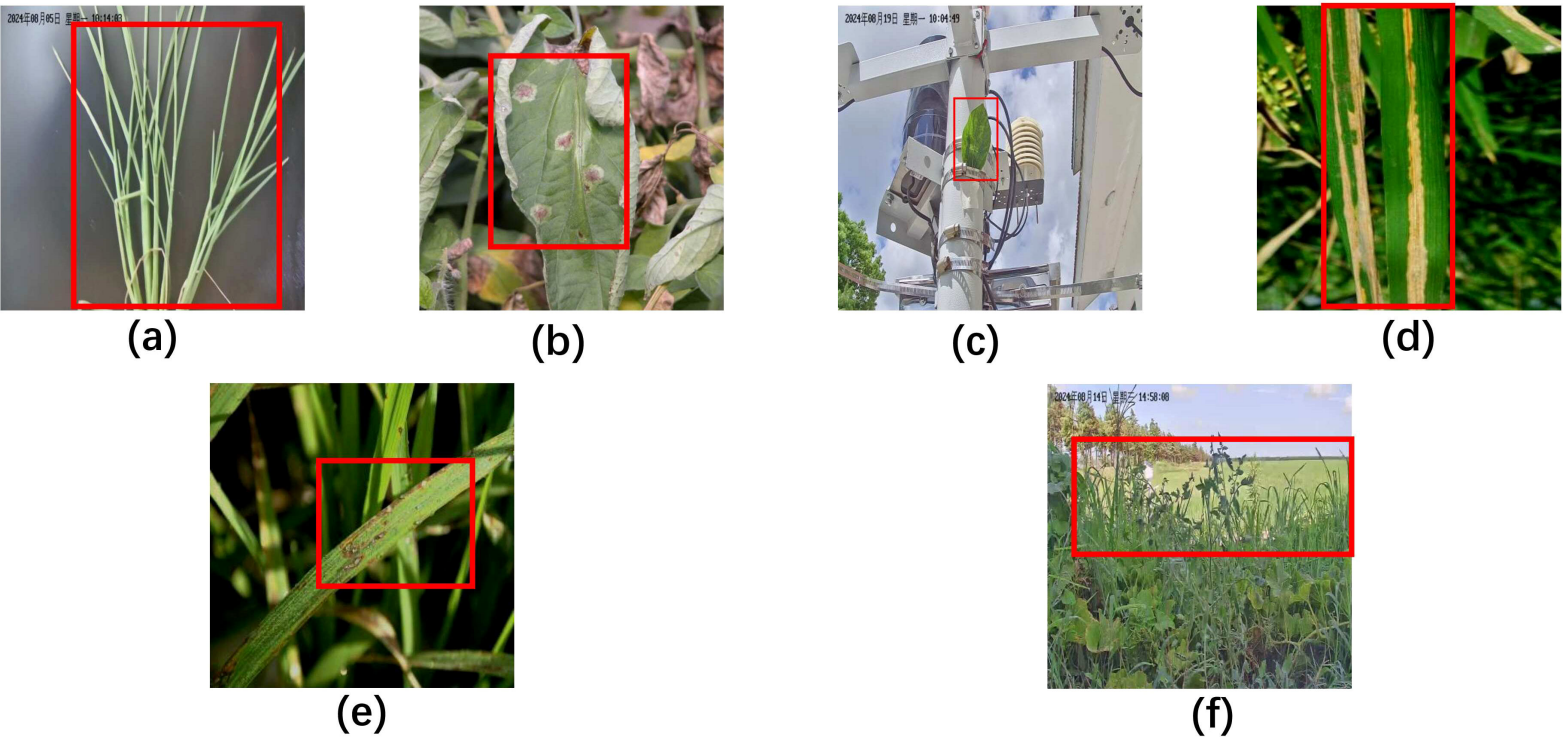
Plant disease types in the RTFD dataset. Each image highlights the disease features with a red box. **(a)** HealthRiceLeaf; **(b)** TomatoLateBlight; **(c)** HealthTomatoLeaf; **(d)** RiceBacterialBlight; **(e)** RiceBrownSpot; **(f)** HealthSTomatoLeaf.

Following augmentation, the dataset was partitioned into training (1,206 images), validation (343 images), and test (179 images) sets, adhering to an approximate 7:2:1 ratio. All images were manually annotated using the Roboflow tool. The dataset comprises six distinct leaf categories: HealthRiceLeaf, RiceBacterialBlight, RiceBrownSpot, HealthTomatoLeaf, HealthSTomatoLeaf, and TomatoLateBlight, as visually cataloged in **Figure 2**.

**Figure 3:**
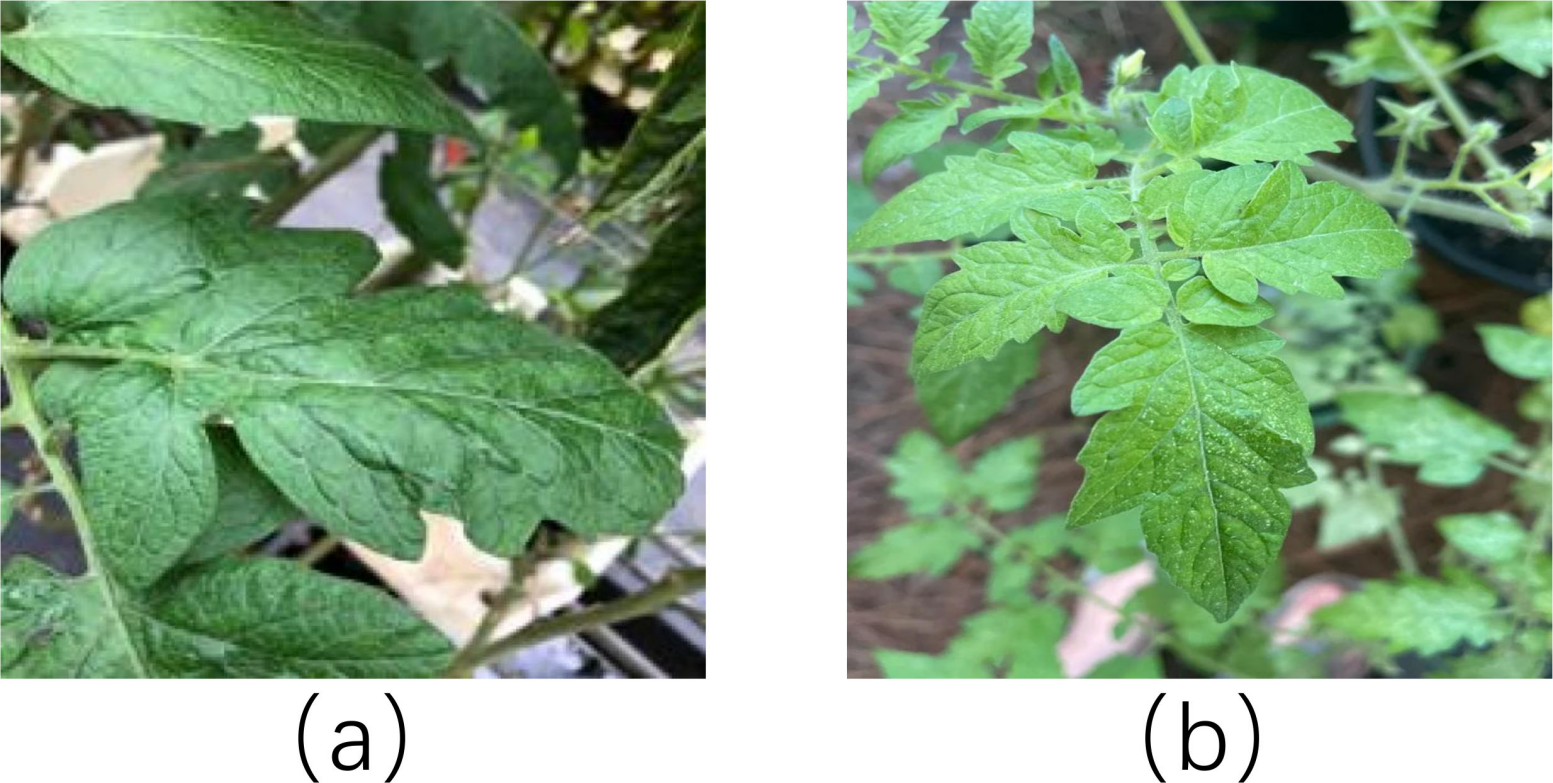
**(a)** HealthTomatoLeaf: larger, broader, and darker in color. **(b)** HealthSTomatoLeaf: smaller, more slender, and lighter in color.

Morphologically, the tomato classes exhibit distinct characteristics. HealthTomatoLeaf (Common Tomato) typically displays a larger surface area with fewer, more obtuse lobes and a high density of trichomes. In contrast, HealthSTomatoLeaf (Cherry Tomato) is typically smaller, characterized by more numerous and sharper lobes, and exhibits a more elongated pinnate morphology. **Figure 3** illustrates the differences between HealthTomatoLeaf and HealthSTomatoLeaf. Regarding pathology, TomatoLateBlight is characterized by large, water-soaked lesions appearing as dark green areas in early stages, which progress to dark brown necrosis.

For the rice categories, HealthRiceLeaf is typically long and slender with a smooth, uniform vibrant green surface. Pathologically, RiceBacterialBlight manifests as water-soaked streaks along leaf margins; these appear as yellowish lesions initially, turning grayish-white and causing desiccation as the disease advances. RiceBrownSpot is distinguished by small, oval-to-circular brown spots with grayish centers and reddish-brown margins, which may coalesce in severe cases.

As detailed in **Table 1**, the instance distribution reflects a significant natural class imbalance, mirroring the stochastic nature of real-world phytopathological occurrences. To accurately reflect authentic agricultural scenarios, the dataset preserves this natural ecological distribution rather than enforcing an artificial balance. For example, high-density categories like HealthRiceLeaf account for 26.98% of the annotations, whereas minority classes such as HealthSTomatoLeaf comprise only 6.56%. Furthermore, the overall instance ratio of diseased lesions to healthy leaves is maintained at approximately 1.47:1 (3,721 diseased vs. 2,528 healthy instances). We deliberately refrained from employing heuristic data-level balancing strategies, such as oversampling, to prevent the model from overfitting to synthetic duplicates and to provide a rigorously realistic benchmark for evaluating model robustness under actual field priors. (The algorithmic mitigation of this imbalance via dynamic loss weighting is detailed in Section 5). To further evaluate the generalization capability of FD-DEIM beyond our primary dataset, we next introduce the publicly available PlantDoc dataset.

**Table 1:** Composition and distribution of each disease of the RTFD dataset.

Disease category	Training	Validation	Test	Total	Proportion
HealthRiceLeaf	1090	410	186	1686	26.98%
TomatoLateBlight	679	400	154	1233	19.73%
HealthTomatoLeaf	244	125	63	432	6.91%
RiceBacterialBlight	721	350	94	1165	18.64%
RiceBrownSpot	1140	139	44	1323	21.17%
HealthSTomatoLeaf	323	43	44	410	6.56%
Total	4197	1467	585	6249	100%

#### 3.1.2 The PlantDoc benchmark dataset

To assess the generalization performance of FD-DEIM, we utilized the public PlantDoc dataset (Singh et al., 2019). This dataset is a widely recognized benchmark known for its ecological realism, featuring 2,598 images across 13 plant species and 31 distinct disease/health categories. Its complexity, characterized by cluttered backgrounds, variable lighting, and diverse lesion morphologies, provides a challenging testbed for evaluating model robustness and scalability beyond the scope of the RTFD dataset.

### 3.2 Methods

**Figure 4:**
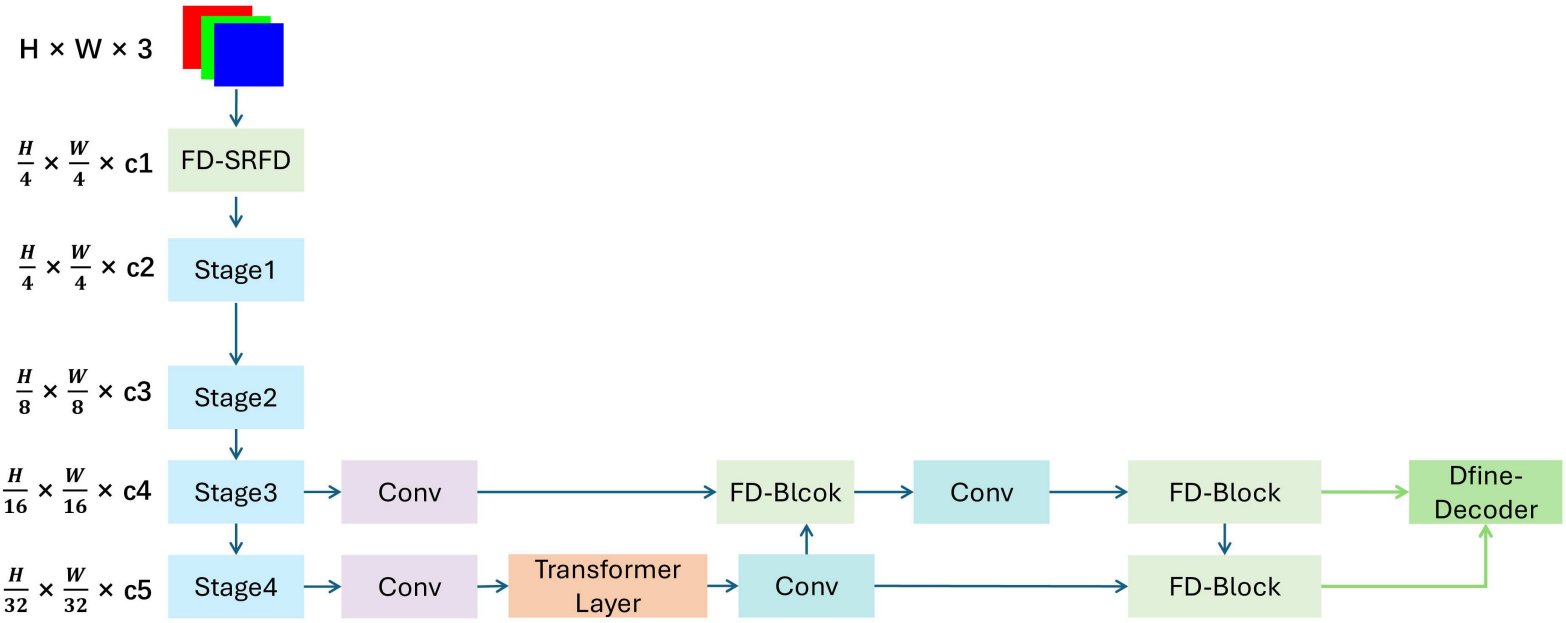
The structure of FD-DEIM.

To address the critical challenges of detecting small, irregular foliar lesions in complex field environments, we propose FD-DEIM, a lightweight and robust detection model. The architecture is engineered upon the DEIM-D-FINE-N baseline (Huang et al., 2025), a powerful real-time detection framework, by integrating three novel components specifically designed to enhance feature preservation and fusion efficiency. The overall structure, depicted in **Figure 4**, follows a standard paradigm comprising a backbone, neck, and head. Our primary innovations are strategically embedded within this framework: the FD-SRFD module replaces the initial stem block in the HGNetv2 backbone, while the DRFD module and FD-Block enhance the downsampling and feature fusion pathways in the neck. This integrated design creates a robust feature extraction pipeline that feeds high-quality, detail-rich information to a shape-adaptive detection head, systematically finish detection task.

#### 3.2.1 FD-SRFD stem module

The initial layers of a network are critical for preserving the fine-grained details necessary for small object detection. Standard stem modules, however, frequently destroy the subtle edge and texture information of early-stage lesions. First, conventional downsampling relies on stride-2 convolutions and max-pooling, acting as a low-pass filter that irreversibly discards high-frequency spatial details. In phytopathology, where lesions often present as microscopic and localized spots, this detail loss directly compromises localization accuracy. Second, standard stems lack the representational capacity to distinguish diverse lesion textures from complex agricultural backgrounds (e.g., varying illumination, leaf occlusions, or soil patterns).

**Figure 5:**
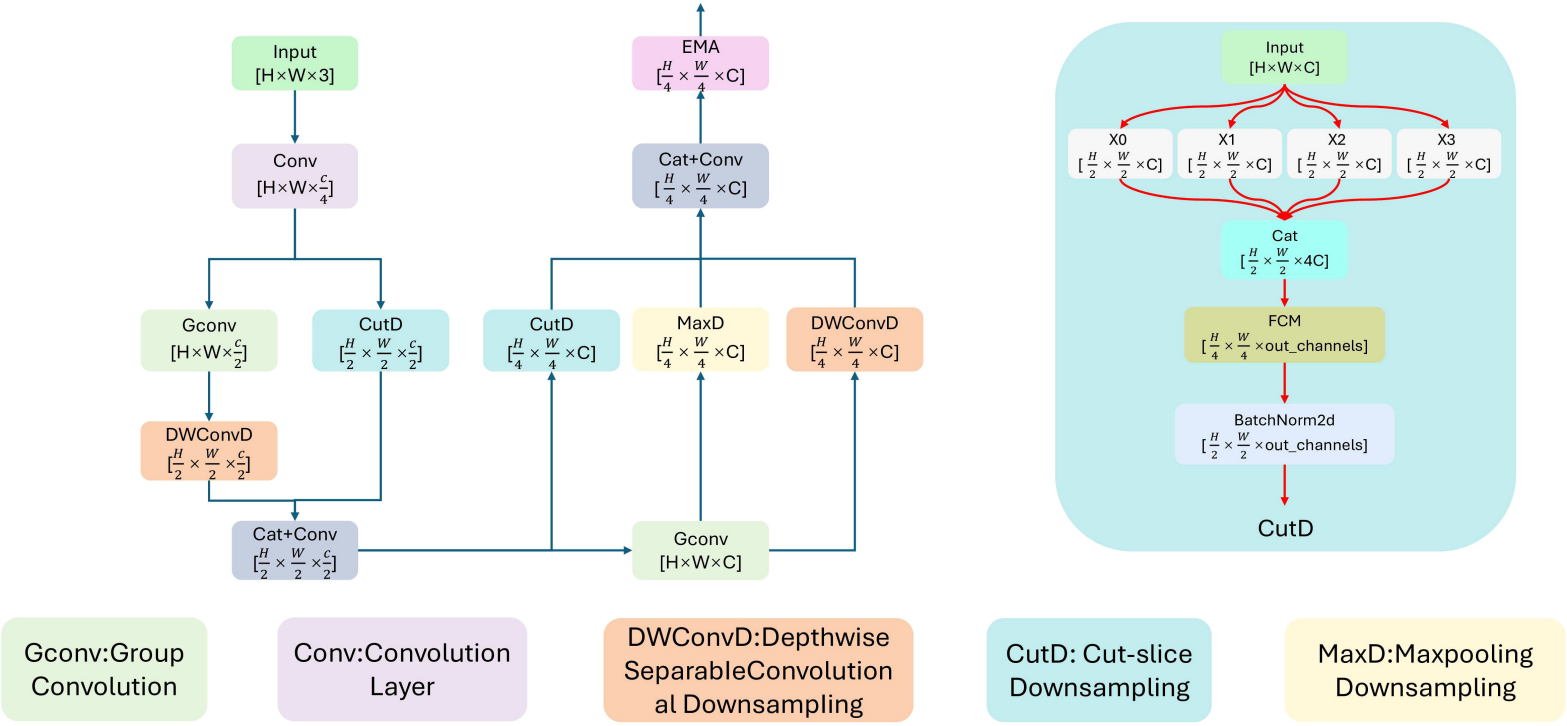
The structure of FD-SRFD module, which combines three submodules: SRFD, EMA, and FCM. It enhances detection accuracy. The black dashed box delineates the baseline SRFD paradigm, while the red solid boxes highlight our specific additions: the EMA mechanism and the FCM module.

To address these structural bottlenecks, we replace the original stem of the HGNetv2 backbone with our proposed FD-SRFD module. This architecture builds upon the foundational Shallow Robust Feature Downsampling (SRFD) mechanism (Lu et al., 2023), which shares the same origin as the DRFD module utilized in our network neck. As illustrated in **Figure 5**, the base SRFD utilizes a multi-path downsampling strategy that processes features in parallel through slicing (CutD), convolution (ConvD), and max-pooling (MaxD) after an initial 7 × 7 convolution to mitigate early information loss. However, while the original SRFD was designed to preserve generic high-frequency signals in remote sensing scenarios, it lacks the specialized inductive biases required to isolate pathological textures from severe agricultural background noise.

Therefore, we elevate this baseline into the FD-SRFD by introducing targeted architectural enhancements specifically optimized for microscopic lesion extraction. Our primary structural improvements within this module are the integration of the Feature Complementary Module (FCM) (Xiao et al., 2025) and the Efficient Multi-Scale Attention (EMA) (Ouyang et al., 2023):

1. FCM for Sliced Spatial Restoration: Within the CutD path, the physical slicing operation inherently disrupts the local spatial contiguity of microscopic lesions, which may span only a few pixels. To compensate, we embed the FCM during feature fusion. FCM explicitly models local context to reassemble these fragmented sub-pixel clues across the sliced channels. This prevents the textural features of tiny spots from being discarded as isolated noise, ensuring that raw pixel fidelity is retained.
2. EMA for Coordinate-Preserving Attention: At the module’s entrance, we deploy the EMA mechanism. Our motivation for selecting EMA over conventional attention mechanisms (such as SE or CBAM) stems from the specific demands of in-field disease detection. Standard attention models rely heavily on Global Average Pooling (GAP), which irreversibly dilutes the localized activation of microscopic lesions into the dominant background signal of large field images. EMA bypasses this limitation by utilizing cross-spatial learning without drastic spatial dimension reduction. This joint channel spatial filtering effectively suppresses non-target textures—such as leaf veins, specular reflections, and soil—while strictly preserving the fine-grained spatial coordinates of true lesion areas.

The result is a highly discriminative and detail-rich feature representation passed to the deeper layers of the backbone, establishing a solid foundation for detecting small, irregular targets.

#### 3.2.2 Multi-scale feature integrity

The neck of a detector is responsible for fusing features from different scales to build a comprehensive scene representation. This is vital for detecting lesions that can vary significantly in size. We introduce two key innovations in the neck: the DRFD module for downsampling and the FD-Block for feature fusion.

##### 3.2.2.1 A. DRFD

**Figure 6:**
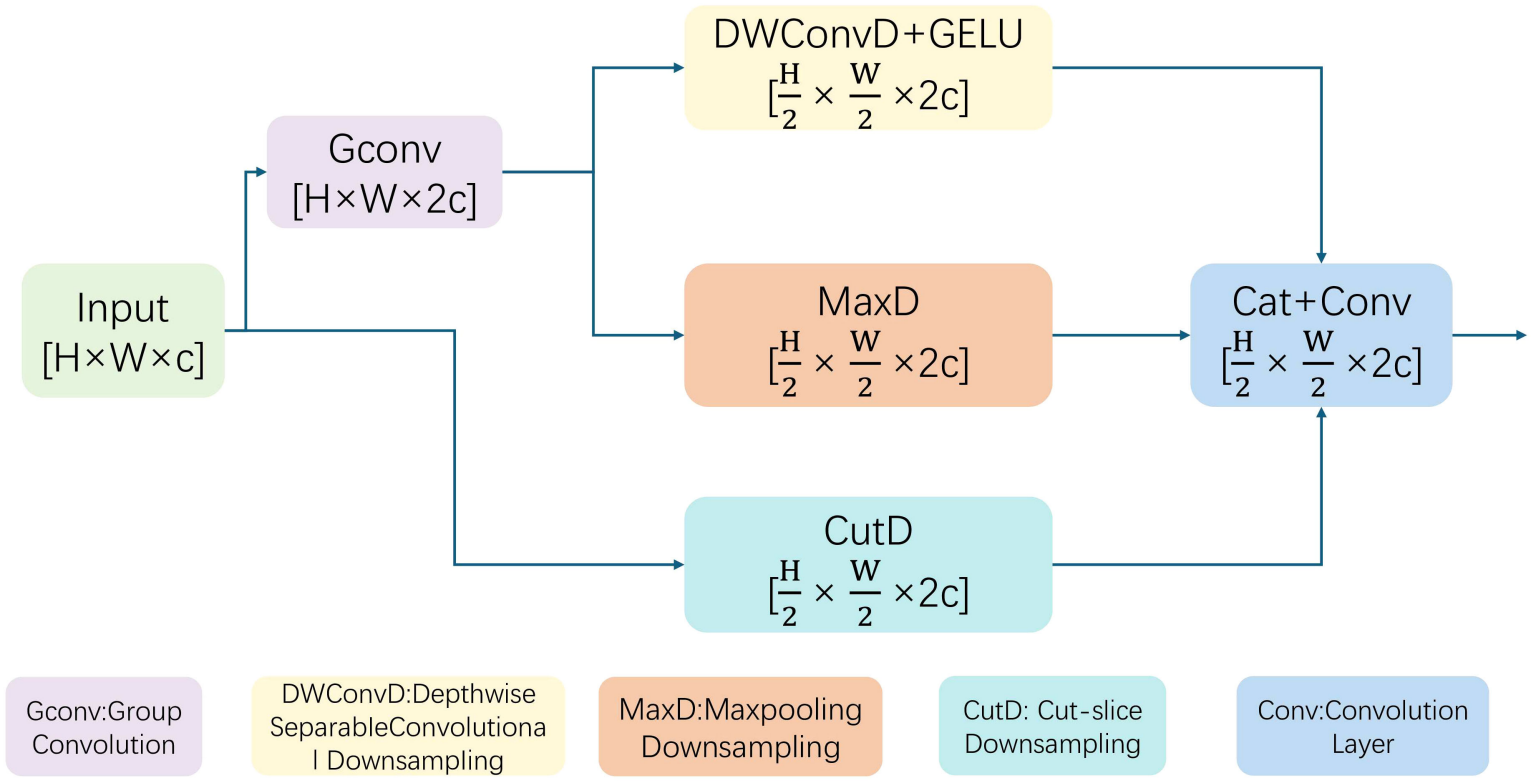
The structure of the DRFD module, which combines three parallel downsampling paths to preserve feature details.

Within the feature pyramid, standard downsampling operators often introduce aliasing artifacts and information loss, which is particularly detrimental to the boundaries of small, irregular lesions. We replace the conventional SCdown module—the standard downsampling operator used in the baseline’s neck—with the Deep Robust Feature Downsampling (DRFD) module (**Figure 6**). The DRFD module employs a triple path architecture to ensure feature integrity by concurrently using three parallel downsampling techniques: slicing-based (CutD) to preserve original pixel information, pooling-based (MaxD) to emphasize salient features, and convolution-based (ConvD) for local feature fusion.

Specifically, the channels are equally distributed among the three paths (CutD, ConvD, and MaxD) with a ratio of 1:1:1 before the final fusion. The ConvD path utilizes a 3 × 3 depthwise convolution followed by a stride-2 3 × 3 depthwise convolution to capture local hierarchical features, while the MaxD path employs a 2 × 2 max-pooling operation to extract salient structural cues. These parallel outputs are concatenated and integrated via a 1×1 convolution layer to aggregate multi-perspective spatial information. By integrating these diverse perspectives, DRFD creates a more robust and complete feature representation during resolution reduction, ensuring that critical edge and low-contrast chromatic details of lesions are maintained.

##### 3.2.2.2 B. FD-Block

Standard architectures employ Feature Pyramid Networks (FPN) (Lin et al., 2017) and Path Aggregation Networks (PAN) (Liu et al., 2018) to fuse semantic and spatial information via top-down and bottom-up pathways. In our baseline, the RepNCSPELAN4 block manages this fusion. Despite its multi-branch structure, RepNCSPELAN4 relies on standard convolutions, which limits feature diversity and hampers generalization across varying illumination angles or plant species. Furthermore, the stacking of multiple VGGBlocks creates excessive computational overhead. This high parameter count impedes deployment on resource-constrained agricultural edge devices. To overcome these limitations, we propose the FD-Block, which redesigns the feature fusion mechanism through three key strategies. First, it integrates the CSP structure of the C2f Block to mitigate information loss from strided convolutions. By employing channel splitting, this design preserves fine-grained texture details essential for identifying small lesions. Second, we incorporate the Faster Block (Chen et al., 2023), which utilizes partial convolutions (PConv) and MLPs to extract diverse spatial and channel features. This enhances robustness against environmental variance similar to the DRFD module. Notably, PConv reduces redundancy through group processing ( n_div), while DropPath and LayerScale regularization prevent overfitting. This ensures high efficiency for real-time diagnostics without compromising multi-scale detection accuracy.

**Figure 7:**
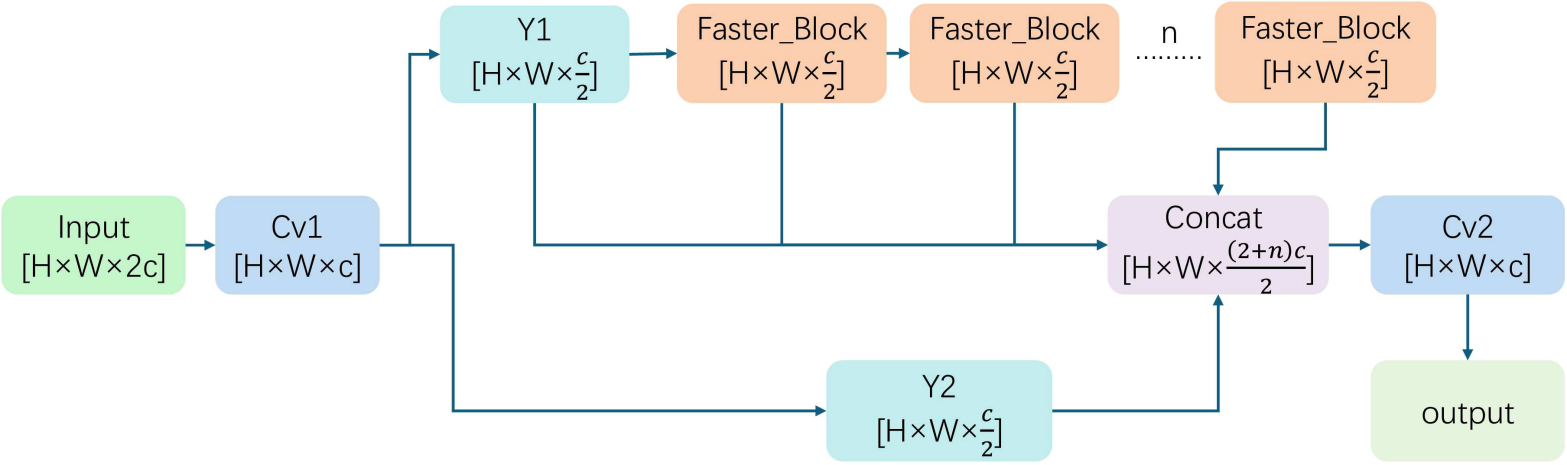
The structure of FD-Block module, which combines C2f-block and Faster-Block to make the model lightweight.

The diagram of the FD-Block model is as follows **Figure 7**, and the feature fusion is calculated by Equation 1.

(1) $$Y=Cv2(Concat(Y_{2},Z_{0},Z_{1},\ldots ,Z_{n}))$$

where (*Y*_1_*, Y*_2_) represents the output of a channel-wise split operation applied to Cv1(X), $Z_{0}=Y_{1}$, and *Z_i_* denotes the output of the *i*-th Faster Block. The parameter *n* signifies the number of Faster Blocks, which is dynamically determined by the network depth scaling factor (*n* = round(3 × depth_mult)). In our proposed lightweight FD-DEIM, *n* is set to 3.

#### 3.2.3 High-precision decoding for irregular lesions

The final challenge in phytopathological detection is accurately localizing lesions with amorphous, non-convex boundaries. Traditional detectors rely on rigid anchor boxes or static grid assignments, which are geometrically misaligned with the organic shapes of fungal infections, leading to suboptimal Intersection over Union (IoU) and compromised boundary precision.

To resolve this structural bottleneck, we integrate the D-FINE decoder (Peng et al., 2024) as our detection head. While D-FINE is an advanced framework originally established for general object detection tasks, our work explicitly leverages its unique architecture to address the specific “rigid-anchor versus irregular-lesion” mismatch in agriculture. By combining this decoder with our custom detail-preserving backbone and neck (FD-SRFD and DRFD), we formulate a highly specialized detection pipeline.

Specifically, the D-FINE decoder employs a dynamic, offset-based sampling mechanism. Instead of sampling features from a fixed grid, it predicts coordinate offsets that dynamically reposition the sampling points to conform tightly to an object’s true contours. By feeding this offset-aware decoder with the high-fidelity, fine-grained feature maps preserved by our front-end pipeline, the network can adaptively align its receptive field with the irregular margins of foliar lesions. This structural integration successfully translates a dynamic regression mechanism into a highly precise localization tool for microscopic agricultural lesions, significantly improving strict bounding box metrics (e.g., AP@75).

## 4 Results

### 4.1 Training parameters and setting

The model was trained in a Linux environment utilizing PyTorch and a GPU. The specific hardware and software configurations are presented in **Table 2**.

**Table 2:** Configuration of the experiment environment.

Item	Configuration
CPU	AMD EPYC 7K62 48-Core Processor
Memory	60GB
GPU	Nvidia RTX 4090D
Debian version	6.1.135-1
PyTorch version	3.0.0

For training on these datasets, the AdamW optimizer was employed with a base learning rate of 1 ×10^−4^ and weight decay of 1 × 10^−4^. The training process spanned 280 epochs and was systematically structured into four stages to ensure optimal convergence. Epochs 0–4 were designated as the first stage, employing a basic data augmentation mode with a linear warmup. Epochs 4–144 were defined as the second stage, during which strong data augmentations were fully activated. Specifically, multi-scale training was implemented within the Mosaic augmentation using a scaling range of [0.5,1.5], a rotation range of 10^°^, and a translation range of 0.1. Mixup augmentation was applied with a probability of 0.5. Additional robust augmentations included Random IoU Crop (*p* = 0.8) and Random Photometric Distort (*p* = 0.5).

Epochs 144–259 constituted the third stage, where Mixup was deactivated to prevent manifold underfitting, while Mosaic and other augmentations were retained. The final stage, spanning epochs 259–280, deactivated all strong spatial distortions, reverting to the basic data augmentation mode to allow the model to fine-tune on the true data distribution at a fixed 640 × 640 resolution. An Exponential Moving Average (EMA) with a decay rate of 0.9999 was also utilized to stabilize the training weights. This stage-wise configuration efficiently supported model convergence without relying on pre-trained weights, effectively extracting diverse features from the limited sample size while mitigating the risk of overfitting.

### 4.2 Performance metrics

To strictly evaluate detection performance and computational efficiency, we adopted standard Microsoft COCO evaluation protocols. The primary accuracy metrics include AP@50 (IoU=0.50), AP@75 (strict boundary precision), and AP@[50:95] (mean AP across IoU thresholds from 0.50 to 0.95). Furthermore, considering the prevalence of microscopic early-stage lesions in the RTFD dataset, we introduced AP*_S_* to specifically quantify detection performance on small objects. Efficiency is measured via GFLOPs and parameter count. The metrics are mathematically defined in Equations 2–5:

(2) $$\text{AP}@50=\frac{1}{N_{cls}}\sum_{c=1}^{N_{\text{cls}}}{\text{AP}}_{c}^{0.50}$$

(3) $$\text{AP}@75=\frac{1}{N_{cls}}\sum_{c=1}^{N_{\text{cls}}}{\text{AP}}_{c}^{0.75}$$

(4) $$\text{AP}@[50:95]=\frac{1}{10}\sum_{k=0}^{9}{\text{AP}}^{0.50+0.05k}$$

(5) $${\text{AP}}_{S}={\text{AP}}^{\text{IoU}}\text{ s}.\text{t}.\;{\text{Area}}_{\text{obj}}<32^{2}$$

where $N_{\text{cls}}$ denotes the number of classes, ${\text{AP}}_{c}^{0.50}$ and ${\text{AP}}_{c}^{0.75}$ represent the average precision for class c at IoU thresholds of 0.50 and 0.75, respectively. AP@[50:95] averages the precision over 10 IoU steps to evaluate overall robustness. AP*_S_* strictly targets small targets with pixel area *<* 32 × 32, serving as a critical indicator for early disease detection capabilities.

### 4.3 Ablation study

**Figure 8:**
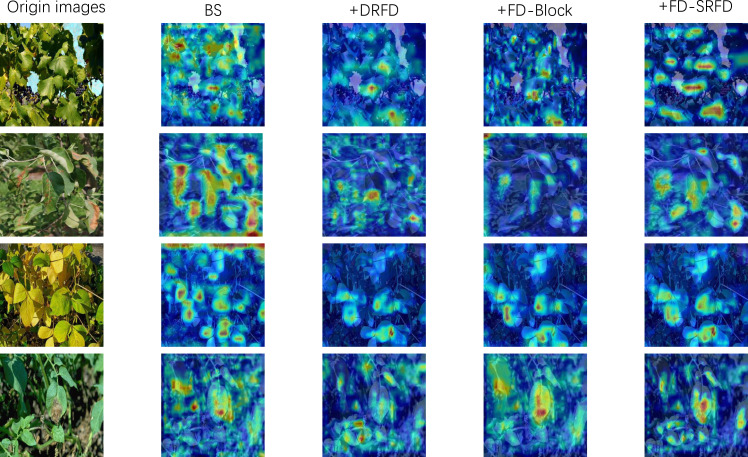
Visualization of ablation study via Grad-CAM heatmaps. The baseline (BS) exhibits scattered attention on the background foliage. With the sequential addition of DRFD, FD-Block, and FD-SRFD, the activation regions progressively shift away from background noise and concentrate intensely on the lesion targets, with FD-DEIM showing the most precise focus.

We performed component-wise ablation experiments on the PlantDoc and RTFD datasets to quantify the impact of the DRFD module, FD-Block, and FD-SRFD stem. Quantitative metrics are detailed in **Tables 3**, **4**, supported by Grad-CAM visualizations in **Figure 8**.

**Table 3:** Ablation experiments of the proposed algorithm on PlantDoc dataset.

Model	AP@50	AP@75	AP@[50:95]	Params (M)	GFLOPs
BS	0.631	0.416	0.389	3.73	7.1217
BS+DRFD	0.647	0.435	0.400	3.82	7.1654
BS+FD-Block	0.512	0.456	0.398	3.67	7.0194
BS+FD-SRFD	0.642	0.459	0.421	3.73	7.6636
BS+DRFD+FD-Block	0.522	0.465	0.408	3.77	7.0630
BS+DRFD+FD-SRFD	0.519	0.467	0.407	3.84	7.7214
BS+FD-Block+FD-SRFD	0.654	0.452	0.416	3.66	7.6163
Ours (FD-DEIM)	0.667	0.457	0.433	3.76	7.6600

^1^The BS denotes DEIM-D-FINE-N.

**Table 4:** Ablation experiments of the proposed algorithm on RTFD dataset.

Model	AP@50	AP@75	AP@[50:95]	Params (M)	GFLOPs
BS	0.631	0.416	0.389	3.73	7.1217
BS+DRFD	0.647	0.435	0.400	3.82	7.1654
BS+FD-Block	0.644	0.447	0.409	3.66	7.0052
BS+FD-SRFD	0.642	0.459	0.421	3.73	7.6636
BS+DRFD+FD-Block	0.652	0.448	0.412	3.76	7.0489
BS+DRFD+FD-SRFD	0.651	0.441	0.411	3.83	7.7073
BS+FD-Block+FD-SRFD	0.654	0.452	0.416	3.66	7.6163
Ours (FD-DEIM)	0.667	0.457	0.433	3.76	7.6600

**Table 4** reveals the distinct cost-benefit profile of each component. The FD-Block emerged as the efficiency driver; it achieved a structural “win-win” by improving AP@50 (+1.3%) while reducing model complexity (Params: -0.07M; GFLOPs: -0.12G) relative to the baseline. The DRFD module demonstrated a high return on investment, delivering a 1.6% AP boost with negligible computational overhead (+0.04 GFLOPs), validating its efficiency in suppressing background noise. Conversely, the FD-SRFD stem introduced a noticeable computational penalty (+0.54 GFLOPs). However, this overhead is structurally necessary: by maintaining high-resolution feature maps, it enables the detection of microscopic lesions that are otherwise lost by aggressive downsampling. Qualitative heatmaps (**Figure 8**) corroborate these metrics, showing that the full architecture generates more concentrated attention on lesion boundaries compared to the baseline.

Regarding initialization strategies, comparisons between training from scratch and initialization using official HGNetv2 pre-trained weights reveal a counter-intuitive advantage for the scratch-trained model (FD-DEIM: 0.540 vs. Pre-trained: 0.520 AP@50). We trace this phenomenon to the interaction between the lightweight architecture and the specific training dynamics of the DEIM framework. Unlike standard detectors, DEIM relies on the Dense O2O strategy, which synthesizes dense supervisory signals via aggressive stochastic augmentations (specifically Mosaic and Mixup) (Huang et al., 2025). For a lightweight backbone with limited capacity, reconciling the rigid, object-centric priors of ImageNet pre-training with these spatially distorted, multi-target inputs creates conflicting gradient directions, inducing severe optimization volatility. Training from scratch avoids this interference, allowing the network to adapt flexibly to the Dense O2O distribution. Furthermore, the inherent stochasticity of these dense augmentations introduces variance into the optimization trajectory, causing the model to settle into slightly diverse local minima across runs. This necessitates the use of mean ± standard deviation metrics (**Tables 5**, **6**) to rigorously validate system reliability rather than relying on peak epoch scores.

**Table 5:** Comparative experiments on PlantDoc dataset.

Models	AP@50	AP@75	AP@[50:95]	Params (M)	GFLOPs
Ours (FD-DEIM)	0.540 ± 0.008	0.497 ± 0.007	0.427 ± 0.006	3.77	7.7
D-FINE-N	0.467 ± 0.002	0.422 ± 0.006	0.366 ± 0.001	3.73	7.1
YOLOv11n	0.496 ± 0.007	0.452 ± 0.002	0.392 ± 0.007	2.59	6.3
YOLOv12n	0.520 ± 0.004	0.474 ± 0.002	0.415 ± 0.008	2.56	6.4
YOLOv13n	0.497 ± 0.007	0.454 ± 0.009	0.394 ± 0.006	2.45	6.2

**Table 6:** Comparative experiments on RTFD dataset.

Models	AP@50	AP@75	AP@[50:95]	APs	Params (M)	GFLOPs
Ours (FD-DEIM)	0.667 ± 0.008	0.457 ± 0.007	0.433 ± 0.006	0.433 ± 0.013	3.77	7.7
D-FINE-N	0.604 ± 0.008	0.390 ± 0.005	0.379 ± 0.006	0.323 ± 0.007	3.73	7.1
YOLOv11n	0.545 ± 0.001	0.339 ± 0.002	0.334 ± 0.007	0.250 ± 0.006	2.59	6.3
YOLOv12n	0.543 ± 0.007	0.348 ± 0.002	0.334 ± 0.007	0.239 ± 0.007	2.56	6.4
YOLOv13n	0.589 ± 0.008	0.384 ± 0.009	0.369 ± 0.006	0.297 ± 0.016	2.45	6.2

Beyond evaluating isolated components, analyzing their integrated performance reveals significant synergistic mechanisms that explain the non-linear metrics observed in **Table 3**. Specifically, the concurrent application of FD-SRFD and DRFD yields a super-additive improvement in strict boundary precision (AP@75), surpassing the algebraic sum of their individual gains. This enhancement stems from establishing a continuous spatial coordinate preservation pipeline across the network. Because AP@75 is highly sensitive to minor bounding box shifts, preserving sub-pixel coordinates is critical. While the FD-SRFD stem anchors these microscopic cues at the network entrance, standard downsampling in subsequent neck layers would normally erode them. Conversely, deploying DRFD in the neck without the front-end FD-SRFD would lack the high-fidelity features necessary for optimal processing. When integrated, the slicing-based CutD path within DRFD actively protects the initial spatial cues provided by FD-SRFD from drifting during deep feature pyramid construction. This mutual reinforcement effectively mitigates sequential localization displacement, leading to the compounded boost in AP@75.

Furthermore, the structural synergy between the FD-SRFD stem and the FD-Block prevents a linear superposition of computational overhead. This efficiency is driven by representational decoupling. The EMA mechanism embedded in FD-SRFD acts as an early-stage filter, suppressing background noise such as soil and specular reflections before deep processing occurs. Consequently, the downstream FD-Block receives highly purified feature maps. Because the FD-Block utilizes Partial Convolutions (PConv)—which compute filters on merely a subset of channels to minimize redundancy—it operates with maximum efficiency on these denoised inputs. This complementary dynamic allows the network to achieve high-fidelity multi-scale fusion without incurring the expected proportional penalty in GFLOPs.

### 4.4 Comparative study

To benchmark FD-DEIM against state-of-the-art lightweight detectors, we conducted comparative experiments on the PlantDoc and RTFD datasets. We selected the YOLO series (v11n, v12n, v13n) and D-FINE-N—the foundational architecture for our internal baseline—as external benchmarks due to their comparable parameter scales (*<* 4M) and widespread adoption in agricultural robotics. For impartial benchmarking, YOLO models were trained using official Ultralytics defaults (AdamW, lr=2.94 × 10^−4^, momentum=0.9) to eliminate manual tuning bias. D-FINE-N and FD-DEIM followed the transformer-optimized protocol detailed in Section 4.1. It is worth noting that, rather than reporting peak epoch results, we present the Mean ± Standard Deviation derived from five independent runs with distinct random seeds, utilizing independent two-sample t-tests to verify statistical significance.

**Figure 9:**
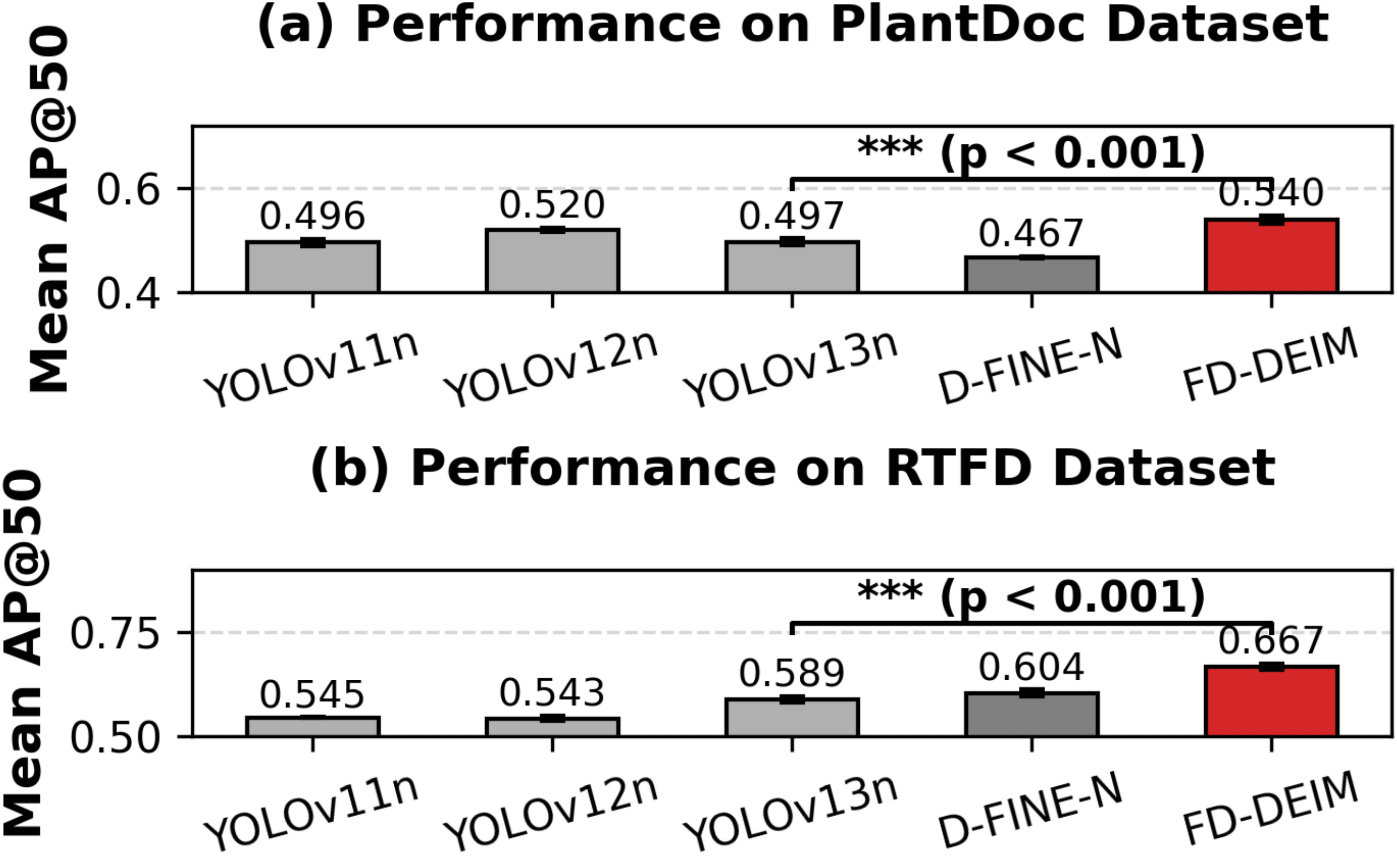
Performance comparison of various detectors using t-test analysis. **(a)** Performance results (Mean AP@50) on the PlantDoc Dataset. **(b)** Performance results (Mean AP@50) on the RTFD Dataset. Independent samples t-tests confirm statistical significance (***p < 0.001) of the differences.

On the PlantDoc benchmark (**Table 5**), FD-DEIM demonstrated superior generalization, achieving a mean AP@50 of 0.540 ± 0.008. This performance significantly outperforms the latest YOLOv13n (0.497 ± 0.007) and the original D-FINE-N (0.467 ± 0.002), with statistical significance confirmed by p-values *<* 0.001 (**Figure 9**). In terms of boundary precision (AP@75), FD-DEIM (0.497) surpassed YOLOv13n by 4.3% and D-FINE-N by 7.5%. These results validate that while D-FINE-N provides a strong baseline, the feature decoupling mechanisms in FD-DEIM are essential for preventing feature collapse on diverse, web-scraped data.

The architectural advantages of FD-DEIM are most pronounced on the ecologically representative RTFD dataset (**Table 6**), which is characterized by a high density of microscopic, early-stage lesions. While the YOLO series plateaued below 0.60 AP@50 (e.g., YOLOv13n at 0.589 ± 0.008), FD-DEIM attained 0.667 ± 0.008. To specifically quantify the capability for early disease detection, we analyzed the small object metric (*AP_S_*). The anchor-free D-FINE-N baseline (0.323 ± 0.007) outperformed the strongest YOLO model (v13n: 0.297 ± 0.016) and significantly eclipsed YOLOv12n (0.239 ± 0.007), exposing the limitations of anchor-based mechanics for small targets. However, FD-DEIM achieved a remarkable *AP_S_* of 0.391 ± 0.013, surpassing D-FINE-N by 21% and YOLOv13n by 31.6%. This substantial margin confirms that the FD-SRFD stem and decoupled blocks effectively retain high-frequency spatial details that are otherwise lost during downsampling.

**Figure 10:**
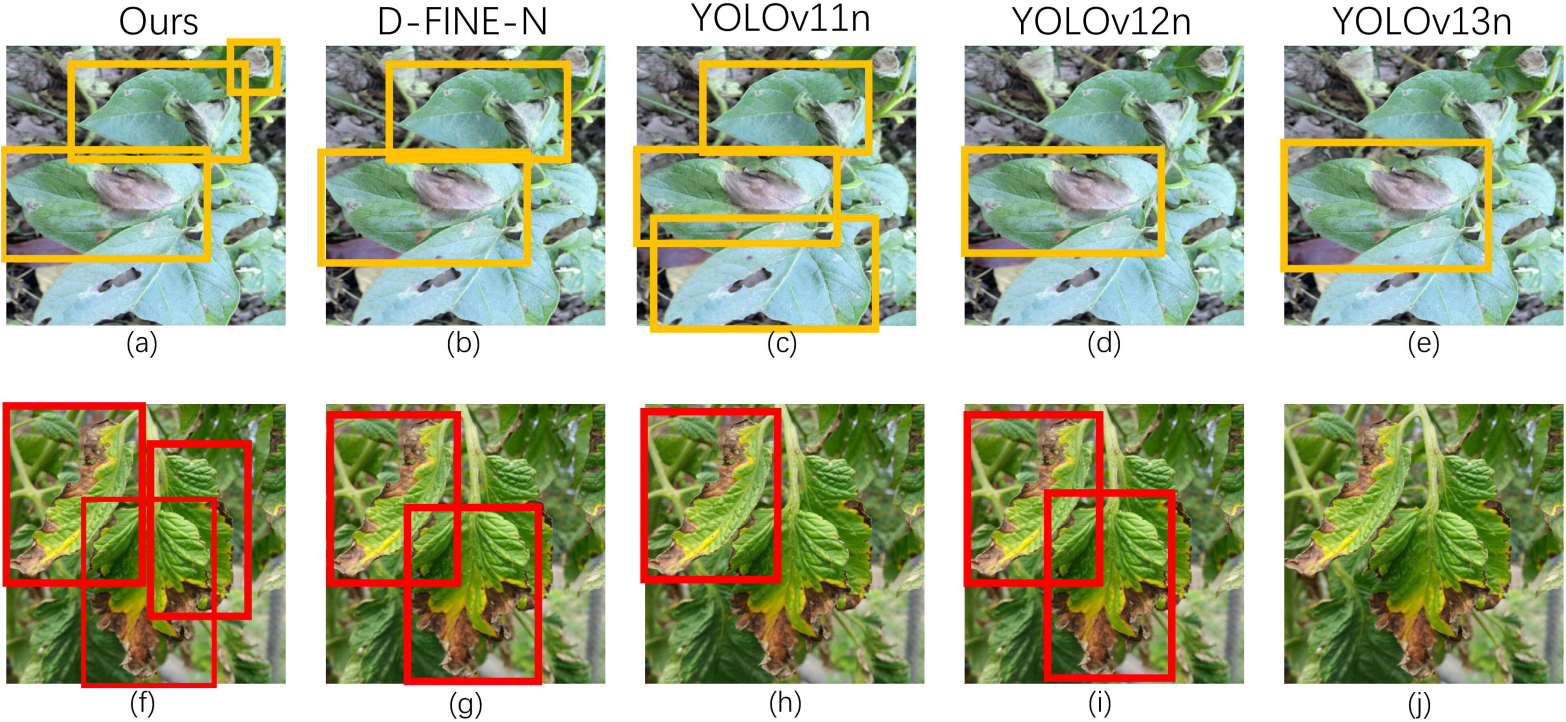
Qualitative comparison on PlantDoc (confidence threshold=0.6). **(b, g)** D-FINE-N shows partial missed detections on subtle lesions. **(e, j)** YOLOv13n fails to output any valid bounding boxes for these irregular targets due to low prediction confidence. In contrast, **(a, f)** FD-DEIM successfully detects and localizes all target lesions.

**Figure 11:**
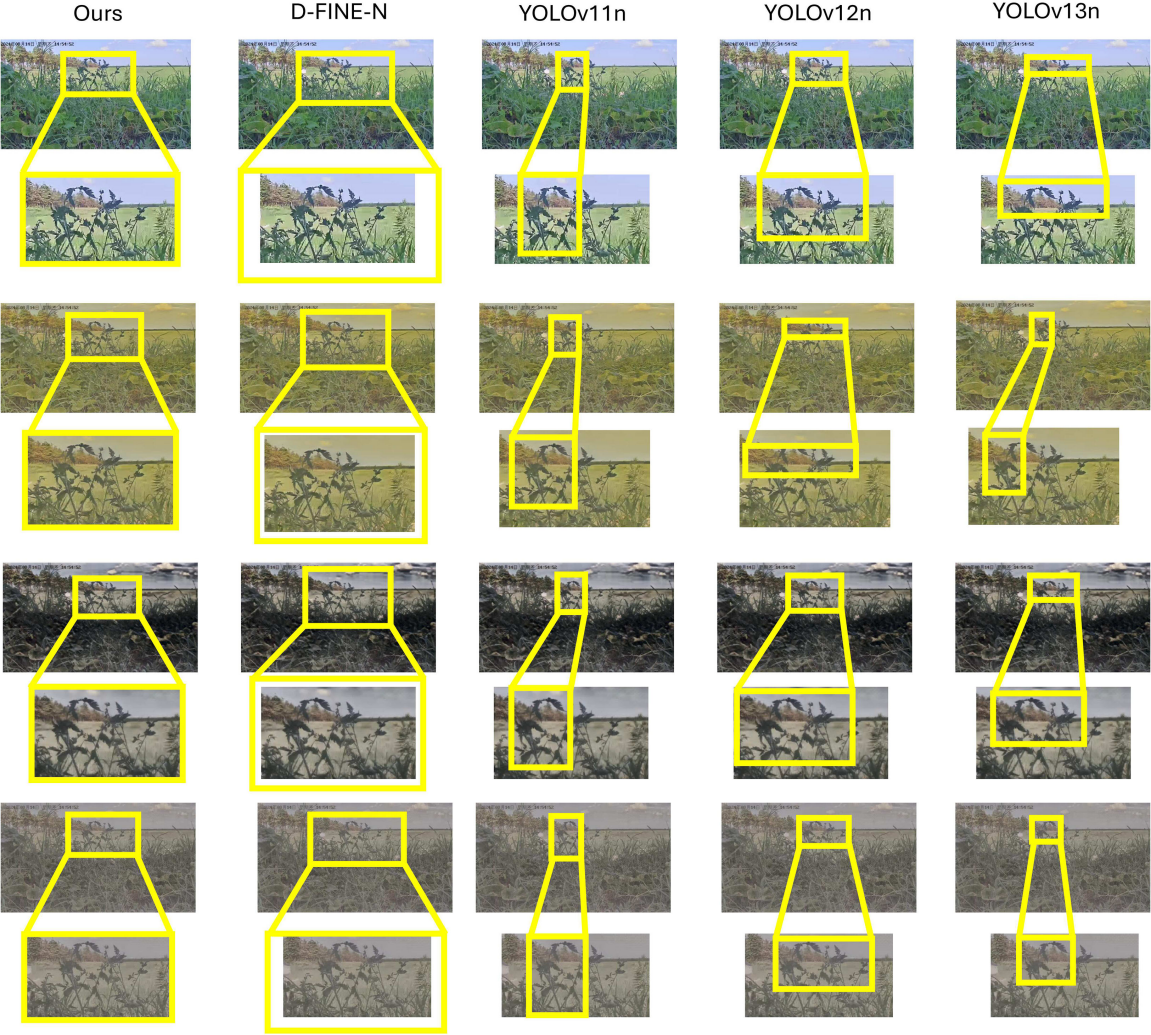
Detection of HealthSTomatoLeaf under varying illumination. Rows represent different weather conditions (e.g., Row 2: High-light). Under strong light interference, baseline models (YOLO series) produce fragmented bounding boxes or fail to cover the leaf tips. FD-DEIM consistently generates complete bounding boxes that encompass the entire leaf structure.

**Figure 12:**
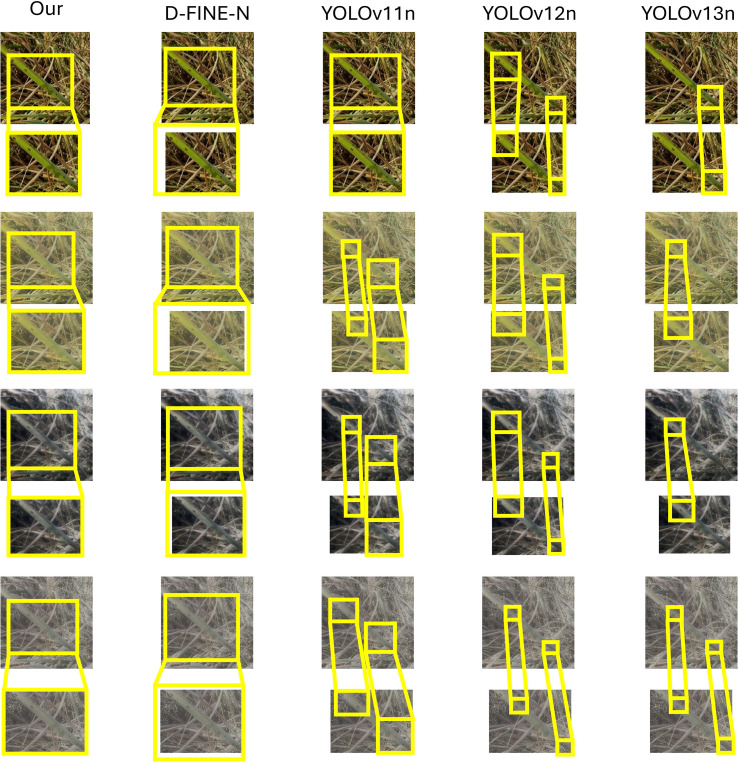
Robustness evaluation on Rice Brown Spot detection. In ideal field conditions (Row 1), all models exhibit comparable detection capabilities. However, under augmented environmental shifts (Rows 2–4), performance divergences become evident. The YOLO series suffers from severe missed detections, failing to capture the complete set of lesions. While D-FINE-N retains detection ability, it exhibits compromised boundary precision (loose boxes). In contrast, FD-DEIM demonstrates superior stability, accurately and completely enclosing all disease spots despite significant environmental interference.

**Figure 13:**
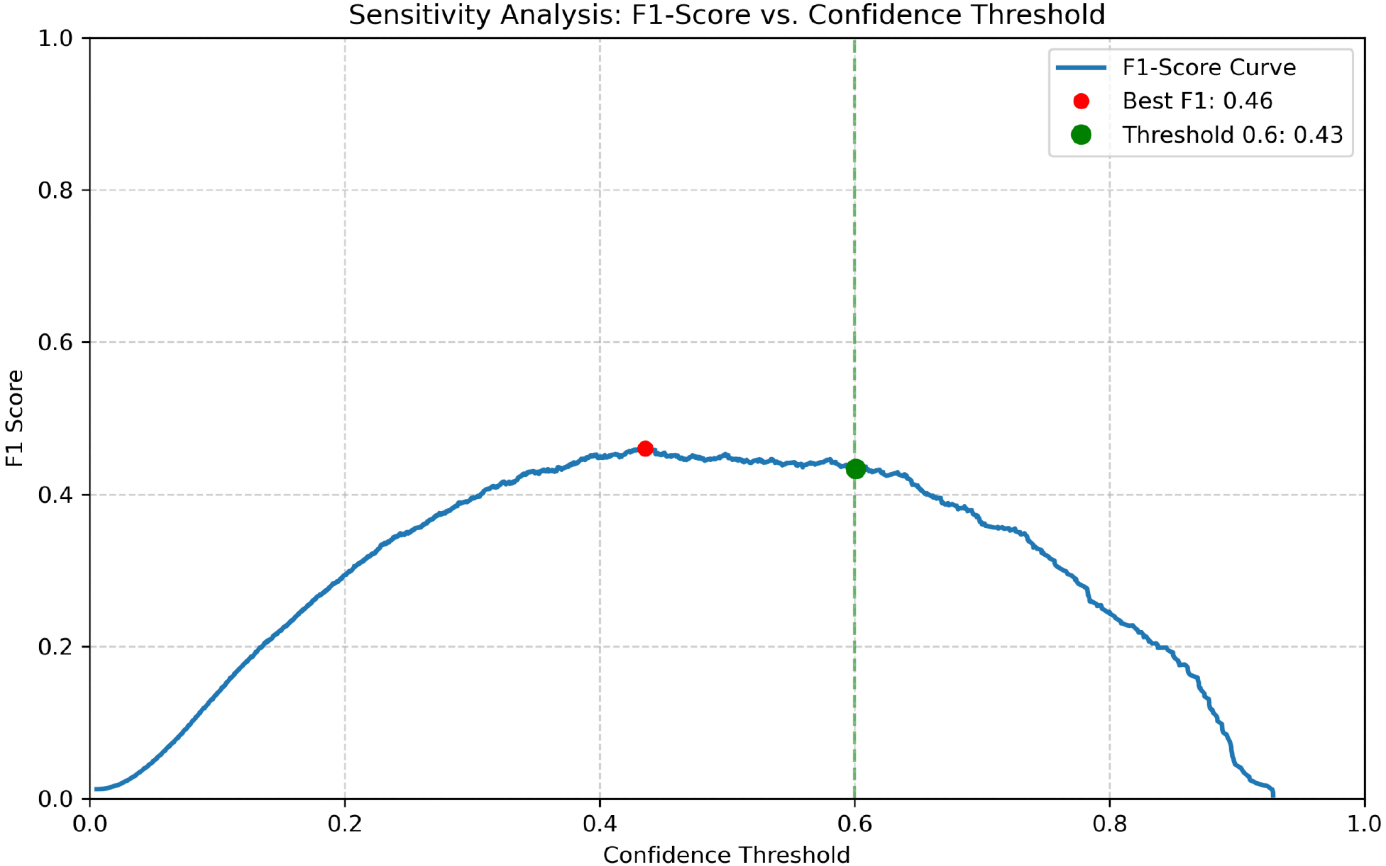
F1-score sensitivity analysis. Although the theoretical peak occurs at 0.43, we selected a stricter threshold of 0.60 (green dashed line) to prioritize precision. The broad stability plateau demonstrates that FD-DEIM maintains robust performance despite strict thresholding, minimizing false positives for field deployment.

Qualitative robustness was further assessed under varying illumination (**Figures 10**–**12**) using a strict confidence threshold of 0.60. As shown in the sensitivity analysis (**Figure 13**), although the theoretical peak F1-score occurs at 0.43, shifting the operating point to 0.60 minimizes false positives with negligible performance loss (*<* 0.03). This trade-off is critical in precision agriculture to prevent unnecessary chemical interventions. Under these rigorous constraints, baseline models frequently missed subtle lesions or produced fragmented boxes in complex backgrounds. In contrast, FD-DEIM maintained high-confidence localization, demonstrating superior reliability for field deployment.

To comprehensively address the scale variance of foliar lesions and clarify the exact detection lower limit, we conducted a fine-grained evaluation specifically targeting Extremely Tiny lesions (*<* 16 × 16 pixels, area *<* 256). Our analysis reveals that standard metrics often obscure the true difficulty of detecting initial-stage infections. For these extremely tiny targets, the baseline model suffered from severe feature vanishing due to aggressive downsampling, resulting in a staggering Miss Rate (MR) of 86.05% and a False Discovery Rate (FDR) of 20.00%. In contrast, by leveraging the multi-path spatial retention of the FD-SRFD stem, FD-DEIM effectively anchored sub-pixel coordinates. As a result, FD-DEIM doubled the number of successfully detected tiny lesions (True Positives increased from 12 to 24), significantly reducing the Miss Rate to 70.73%. Concurrently, the FDR was further suppressed to 17.24%. This quantitative evidence explicitly defines our model’s robust detection lower limit, proving that FD-DEIM successfully overcomes the typical feature erasure problem associated with microscopic early-stage lesions.

### 4.5 Edge deployment verification

**Figure 14:**
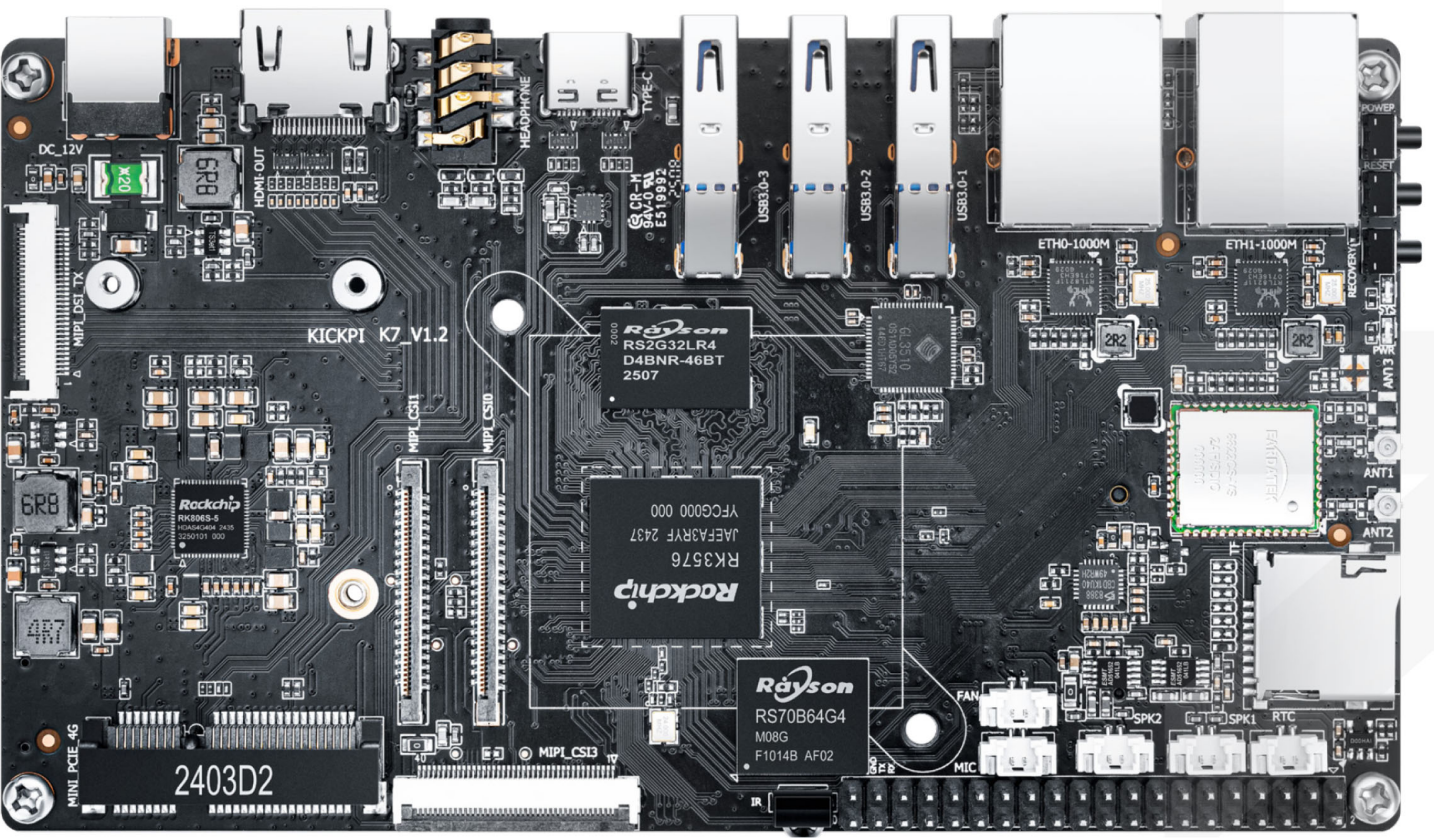
The hardware environment for edge deployment: KICKPI-K7 development board based on Rockchip RK3576 SoC.

To verify the practical applicability of FD-DEIM in real-world agricultural scenarios, we conducted deployment tests on the KICKPI-K7 edge computing platform powered by the Rockchip RK3576 SoC, a high-performance and low-power solution commonly used in agricultural IoT devices (as shown in **Figure 14**).

The hardware specifications are critical for evaluating deployment feasibility. The RK3576 SoC features a heterogeneous octa-core CPU architecture (four Cortex-A72 and four Cortex-A53 cores) and a dedicated Neural Processing Unit (NPU) delivering 6 TOPS of computing power. To facilitate deployment, the trained PyTorch model was exported to the ONNX (Open Neural Network Exchange) format, ensuring compatibility with the edge inference engine.

We evaluated three critical metrics for edge deployment: Model Size (Parameters), Memory Footprint, and Inference Latency. As detailed in **Table 7**, FD-DEIM maintains a compact model size of 14.84 MB, which is highly conducive to storage on resource-constrained devices. In terms of memory footprint, the peak memory usage during inference was recorded at 139.24 MB, well within the typical RAM capacity (4GB/8GB) of commercial edge boards.

**Table 7:** Deployment feasibility analysis and performance comparison of FD-DEIM and baseline models on the Rockchip RK3576 edge platform.

Model	Params (M)	Size (MB)	Memory (MB)	FPS (CPU)	Latency (ms)	AP@50
YOLOv11n	2.59	10.362	134.48	4.09	244.50	0.496
YOLOv12n	2.51	10.336	139.43	3.10	322.58	0.520
YOLOv13n	2.45	9.987	158.69	2.87	348.43	0.497
FD-DEIM (Ours)	3.73	14.840	139.24	3.25	307.69	0.540

Regarding inference speed, we tested the model on the RK3576 CPU to establish a baseline performance. FD-DEIM achieved an inference latency of 307.69 ms per image (equivalent to approximately 3.25 FPS) without dedicated NPU acceleration. While slightly higher than the baseline YOLOv11n due to the introduced complex fusion modules (FD-Block), this latency is acceptable for static field monitoring tasks. Furthermore, considering the RK3576 integrates a 6 TOPS NPU, future quantization (INT8) optimization is expected to increase the inference speed by 5–10 times, fully satisfying the real-time requirements (*>*30 FPS) for drone-based active cruising.

In summary, FD-DEIM demonstrates a superior trade-off: it provides significantly higher detection accuracy with a manageable computational cost, proving its feasibility for edge deployment.

## 5 Discussion

The automated and precise diagnosis of early-stage foliar diseases in complex field environments has long been constrained by two theoretical bottlenecks within standard computer vision architectures: the physical erasure of high-frequency spatial details during downsampling, and the topological misalignment between rigid geometric anchors and the irregular morphologies of biological lesions. While lightweight detectors, epitomized by the YOLO series, have established industry standards for real-time inference, our comparative analysis (**Table 6**) exposes their theoretical limitations when processing fungal infections characterized by stochastic, non-convex boundaries. The proposed FD-DEIM architecture successfully dismantles these structural bottlenecks, establishing a new Pareto frontier between rigorous edge-computing constraints and high-fidelity microscopic detection.

The superiority of FD-DEIM extends beyond conventional global metrics; its true value lies in the quantitative breakthrough in small object detection (*AP_S_*). While the state-of-the-art YOLOv13n stagnated at an *AP_S_* of 0.297, FD-DEIM surged to 0.391, marking a substantial 31.6% leap. Standard convolutional downsampling (e.g., stride-2 max pooling) acts as an aggressive low-pass filter, which inevitably merges the edge features of early bacterial specks into the background leaf texture. Our ablation studies confirm that integrating the FD-SRFD stem with the Deep Robust Feature Downsampling (DRFD) module thoroughly neutralizes this erasure effect. This integration forms a “spatial coordinate preservation chain.” The FD-SRFD stem anchors sub-pixel coordinate cues at the input, while the slicing-based DRFD module protects these high-frequency features from drifting during the construction of deep feature pyramids. Consequently, this unbroken preservation chain explains the super-additive improvement observed in strict boundary metrics (AP@75), as their joint mechanism compounds localization accuracy far beyond the algebraic sum of their individual gains.

Furthermore, the exceptionally high false-negative rate intrinsic to traditional anchor-based models is deeply rooted in their geometric inductive bias: the presumption that targets are rigid, convex polygons. Foliar lesions, such as those in Tomato Late Blight, however, exhibit amorphous and multi-lobed topologies. Forcing orthogonal, axis-aligned bounding boxes to encapsulate diagonally expanding lesions inevitably incorporates excessive background noise, injecting highly destructive label noise into the network during backpropagation. FD-DEIM reconstructs this paradigm by integrating the D-FINE decoder. Moving away from deterministic coordinate regression, it utilizes a Fine-grained Distribution Refinement (FDR) mechanism that allows sampling points to actively deform and tightly hug the organic contours of irregular lesions. This “boundary-aware” capability proves that a probabilistic, dynamic sampling mechanism is structurally optimal for non-rigid targets in precision agriculture.

Beyond architectural topology, our analysis of network optimization dynamics challenges the prevailing reliance on transfer learning in lightweight agricultural detectors. FD-DEIM demonstrated significantly superior generalization when trained from scratch compared to initialization with official ImageNet pre-trained weights (AP@50: 0.540 vs. 0.520). ImageNet weight priors are deeply rooted in object-centric translation invariance. In contrast, the DEIM architecture employs a Dense O2O matching strategy relying on aggressive spatial augmentations (e.g., Mosaic and Mixup), artificially constructing a highly fragmented, dense multi-target supervisory distribution. For a capacity-constrained lightweight network, forcing the reconciliation of rigid pre-trained priors with a severely distorted dense loss landscape triggers optimization volatility. Bypassing pre-training allows the weights to adapt smoothly to the local minima exclusive to the phytopathological data distribution.

A critical data-level challenge accompanying this specific distribution is inherent class imbalance. As reflected in the RTFD dataset, dominant classes (HealthRiceLeaf) significantly outnumber minority classes (HealthSTomatoLeaf). We deliberately rejected heuristic data-level balancing strategies, such as oversampling, to preserve the true prior distribution of the target agricultural environment. Instead, the model’s resilience against this severe long-tail distribution is algorithmically guaranteed at the optimization level by the Varifocal Loss (VFL) (Zhang et al., 2021) integrated into the DEIM framework. By adaptively up-weighting learning signals from minority or hard-to-detect classes while down-weighting overwhelming background samples, VFL effectively neutralizes the negative impacts of class imbalance without distorting the original ecological data distribution.

Ultimately, the practical value of any deep learning model in agriculture depends on its ability to operate within hardware limitations. Our deployment validation on the Rockchip RK3576 heterogeneous edge computing platform provides empirical evidence of FD-DEIM’s viability. The architecture successfully avoids a linear superposition of computational overhead by integrating Partial Convolutions (PConv) (Chen et al., 2023) within the FD-Block. By computing only a subset of feature channels and leaving the rest as identity mappings, PConv filters computational redundancy. This allows the network to process the highly purified inputs from the FD-SRFD stem with maximum efficiency, maintaining high feature diversity while strictly capping the computational load at 7.67 GFLOPs. The resulting 307 ms inference latency on a CPU is entirely sufficient for static field monitoring.

By accurately localizing microscopic infections (*<* 16 × 16 pixels) before macro-visual symptoms spread, FD-DEIM facilitates a paradigm shift from “reactive chemical application” to “proactive precision intervention”. In future work, we aim to expand the temporal span of the RTFD dataset to capture multi-year epidemiological variations and explore INT8 network quantization to compress the FD-DEIM architecture for ultra-low-power microcontroller nodes, further advancing sustainable agricultural ecology.

## 6 Conclusions

This paper proposes a novel model, FD-DEIM, designed specifically for plant disease detection, built upon the DEIM architecture (Huang et al., 2025). To address challenges such as the similarity of plant disease lesions, complex backgrounds, and high recognition difficulty, the model introduces several innovations. Firstly, the DRFD module replaces the original downsampling convolution, effectively reducing background noise. Secondly, the FD-Block module, which integrates C2f-Block and Faster-Block (Chen et al., 2023), minimizes information loss while reducing the overall computational complexity of the model. Thirdly, the FD-SRFD module, tailored to the characteristics of plant disease, combines EMA (Ouyang et al., 2023) with SRFD to replace the original stem module, significantly enhancing detection accuracy and preserving more image details at the backbone’s input. Additionally, a new dataset, RTFD, is proposed. FD-DEIM demonstrates promising performance on both the RTFD and PlantDoc datasets (Singh et al., 2019).

In future research, we will expand the RTFD dataset to enhance its practical application value. To address real-world operational scenarios, we will design specialized operators tailored for the DEIM architecture to fully utilize the Neural Processing Unit (NPU) acceleration capabilities of edge computing platforms, thereby ensuring efficient deployment and validating its practical utility.

## Data Availability

The original contributions presented in the study are included in the article/supplementary material. Further inquiries can be directed to the corresponding author.
